# Underdiagnosed and Undertreated: Obesity and Its Cardiometabolic Burden in Swedish Clinical Practice—Insights From the AROS Database

**DOI:** 10.1111/dom.70845

**Published:** 2026-05-13

**Authors:** Viveca Ritsinger, Magnus Sundbom, Jonatan Dereke, Åsa Ericsson, Thomas Cars, Maria K. Svensson, Anna Norhammar

**Affiliations:** ^1^ Division of Cardiology, Department of Medicine Solna Karolinska Institute Stockholm Sweden; ^2^ Department of Research and Development Region Kronoberg Växjö Sweden; ^3^ Department of Surgical Sciences Uppsala University Uppsala Sweden; ^4^ Novo Nordisk Scandinavia AB Malmö Sweden; ^5^ Sence Research AB Uppsala Sweden; ^6^ Department of Medical Sciences, Renal Medicine Uppsala University Uppsala Sweden; ^7^ Uppsala Clinical Research Centre Uppsala Sweden; ^8^ Capio S:T Görans Hospital Stockholm Sweden

**Keywords:** antiobesity drug, cardiovascular disease, cohort study, obesity therapy, observational study, real‐world evidence

## Abstract

**Aims:**

To quantify real‐world diagnosis and treatment of obesity in Sweden, describe cardiometabolic comorbidity burden across obesity classes, and compare long‐term cardiovascular outcomes with the general population.

**Materials and Methods:**

This population‐based cohort study used the AROS *(Analysis of Real‐world data of patients with Obesity in Sweden)* database to identify adults with a recorded BMI ≥ 30 kg/m^2^ between January 2013 and June 2023. Individuals were stratified by obesity class. Baseline demographics, comorbidities, medications and laboratory values were described. Outcomes included recorded obesity prevalence, obesity diagnosis (ICD‐10: E66), healthcare setting at first BMI ≥ 30 kg/m^2^, cardiometabolic comorbidity profiles, treatment patterns and long‐term cardiovascular outcomes. Cardiovascular outcomes were compared with a matched general population.

**Results:**

In 2022, recorded obesity prevalence was 13.6%. Amongst 328 094 individuals with obesity (mean age 53.5 years; 55.0% women), 67.6% had at least one cardiometabolic comorbidity. At the first observed BMI ≥ 30 kg/m^2^ (index), 28.8% had a recorded obesity diagnosis, increasing to 48.0% 5 years later. Index BMI was most often recorded in primary care (39.7%). Within 5 years after index, 7.8% had received obesity‐management medication and 4.2% had undergone bariatric surgery. Compared with matched population representatives, the obesity cohort had higher cumulative incidence across all cardiovascular outcomes, with the largest relative difference for heart failure hospitalisation (HR 2.34, 95% CI 2.29–2.40).

**Conclusions:**

Obesity remains underdiagnosed and undertreated in Swedish healthcare, despite a high burden of cardiometabolic comorbidities and substantially higher long‐term cardiovascular risk compared with the general population.

## 1 Introduction

Obesity, defined as a body mass index (BMI) ≥ 30 kg/m^2^, is a fast‐growing public health problem. Survey‐based prevalence numbers of obesity are increasing rapidly in most EU countries, ranging between 8% and 24% in 2022 [1]. In Sweden, the prevalence of self‐reported obesity in individuals aged 16–84 years has increased from 11% in 2004 to 18% in 2024 [2]. Whilst obesity is highly prevalent and associated with increased risk of chronic diseases, it is often overlooked and underdiagnosed in daily clinical practice.

Obesity is a major contributor to the population disease burden, largely through its strong association with cardiometabolic comorbidities such as Type 2 diabetes, chronic kidney disease (CKD) and cardiovascular disease. Most individuals with obesity have at least one comorbidity, and the prevalence increases with higher obesity classes [3, 4]. Despite the well‐documented health consequences, obesity remains undertreated in clinical practice, even though randomised trials and real‐world studies consistently demonstrate the efficacy, safety and tolerability of available anti‐obesity therapies [5, 6].

Recognising obesity as a chronic and treatable disease is an important step towards improving clinical outcomes. Greater awareness amongst both healthcare professionals and patients can facilitate timely identification and management. Routine recording of BMI and the inclusion of obesity diagnoses in electronic health records have been associated with higher rates of obesity‐specific care and more successful weight management [5, 7, 8]. However, there is limited up‐to‐date real‐world evidence on the extent of recognition and management of obesity amongst those with high BMI in routine clinical practice [5, 7, 9, 10].

The aim of this large, observational cohort study was to assess the prevalence of obesity in Sweden and to examine the extent of formal diagnosis of obesity across different BMI levels. In addition, we investigated the cardiometabolic comorbidity profiles, treatment patterns and clinical outcomes across obesity classes. We further contextualised the clinical outcomes by comparing them to those of the general population.

## 2 Methods

Sweden's healthcare system is primarily publicly funded through general taxation and universally accessible, ensuring comprehensive coverage for all residents. In addition, each resident's healthcare interactions, including diagnoses, treatments and prescriptions, are recorded using a unique personal identification number, enabling extensive data collection across the population [11].

The study was conducted following the Declaration of Helsinki and was approved by the Swedish Ethical Review Authority (approval numbers 2022‐00638‐01 and 2022‐03382‐02). Given the nature of the study, informed consent was not required.

### 2.1 Data Source

The study utilised data from individuals in the AROS (Analysis of Real‐world data of patients with Obesity in Sweden) database, which links data from National Patient Register, the Swedish Prescribed Drug Register, the National Cause of Death Register, Statistics Sweden (SCB) and regional healthcare data warehouses and electronic health records (EHRs) from Stockholm, Skåne and Dalarna regions [12, 13, 14]. These combined sources include information on diagnoses, prescriptions, clinical and laboratory measurements and mortality, covering a healthcare catchment area representing approximately 40% of the Swedish population. Data were linked via each individual's personal identification number and pseudonymised.

### 2.2 Study Cohorts

Initially, a cohort of patients with obesity (*Obesity cohort*) was defined, comprising all individuals aged ≥ 18 years with at least one recorded BMI ≥ 30 kg/m^2^ between 1 January 2013 and 30 June 2023 from the AROS database. The index date was defined as the first instance in which a BMI ≥ 30 kg/m^2^ was observed for each individual. At least 1 day of follow‐up after the index date was required for inclusion. Participants were stratified by obesity class: class 1 (30.0–34.9 kg/m^2^), class 2 (35.0–39.9 kg/m^2^) and class 3 (≥ 40.0 kg/m^2^).

To contextualise outcomes, a *General population cohort* was established by matching individuals from the Swedish population (3.3 million individuals aged ≥ 18 years in Stockholm, Skåne and Dalarna regions) 1:5 to those in the obesity cohort by year of birth, sex and region of residence, using replacement (i.e., one control could serve as a match for multiple cases). No restrictions on BMI or obesity diagnosis were set for individuals in the *General population cohort*, and therefore may include individuals with overweight or obesity. Each individual in the general population cohort was assigned the same index date as the corresponding matched case.

### 2.3 Patient Characteristics

Demographic information (age, sex and region of residence) was obtained from SCB. Comorbid conditions were identified from the National Patient Register, regional data warehouses and EHRs. We included diagnoses and procedures recorded in primary, specialised outpatient or inpatient care, in any diagnosis position, up to and including the index date. Information on medical treatments was retrieved from the Swedish Prescribed Drug Register, with medication use considered only if dispensed within the 365 days preceding or on the index date. Laboratory values and clinical measurements were extracted from EHRs, using the measurement closest to the index date within a 3‐year look‐back window. For each individual in the *Obesity cohort*, the number of cardiometabolic comorbidities was calculated. Cardiometabolic comorbidities were adopted from commonly used frameworks in obesity and metabolic research and included 10 clinically manifest conditions: hypertension, atrial fibrillation, ischaemic heart disease, heart failure, stroke, peripheral artery disease, dyslipidaemia, diabetes mellitus, CKD and obstructive sleep apnoea [15]. Diagnosis codes (ICD‐10, *International Classification of Diseases, 10th Revision*), clinical procedure codes, drug codes (ATC, *Anatomical Therapeutic Chemical Classification System*), clinical measurements and laboratory values were used to define the cardiometabolic comorbidities. Detailed definitions and code lists are provided in the S1.

### 2.4 Outcomes

Study outcomes included measures of recorded obesity prevalence, cardiometabolic profiles and treatment patterns, as well as clinical outcomes. Clinical outcomes comprised all‐cause mortality, major adverse cardiovascular events (MACE), cardiovascular death, hospitalisation for heart failure, myocardial infarction and stroke. MACE was defined as a composite of cardiovascular death, myocardial infarction and stroke. All outcomes were derived from national and regional healthcare registers and assessed from the index date until the occurrence of death (if not the outcome of interest) or until the end of follow‐up (30 June 2023), whichever came first.

### 2.5 Statistical Analysis

Baseline characteristics were compared across the groups, where continuous variables were reported as mean with standard deviation (SD) or median with interquartile range (IQR), and categorical variables were summarised as absolute numbers and percentages. Prevalence of obesity was estimated from each individual's first recorded indication of obesity, defined as a BMI ≥ 30 kg/m^2^, an obesity diagnosis (ICD‐10 code E66), a dispensed prescription for obesity‐management medications (OMMs) (ATC codes A08AB01, A08AA62 and A10BJ02 [restricted to preparations indicated for obesity treatment]), or a record of bariatric surgery (procedure code JDF). Individuals were then carried forward as categorised with obesity until death or the end of follow‐up. Prevalence was calculated by age group (18–44, 45–64, 65–74, 75–84 and ≥ 85 years), using the number of residents in Sweden on 31 December of each year as the denominator (Statistics Sweden, SCB). To ensure consistent data coverage across regions, the analysis period was restricted to 2017–2022. Although BMI data in the AROS database were available from 2013, coverage of BMI recordings varied between regions and over time, likely reflecting differences in the implementation of structured electronic documentation. Data for 2023 were excluded from the prevalence analysis as the calendar year was not complete at the time of data extraction and therefore did not allow for full‐year prevalence estimation.

The number of cardiometabolic comorbidities (0, 1, 2, 3, or ≥ 4) was summarised for individuals in the *Obesity cohort* and stratified by obesity class. Because age and sex distributions differed across groups, we applied direct age–sex standardisation. For all analyses of comorbidity burden, including comparisons across obesity classes and across healthcare settings, the age (5‐year age groups) and sex distribution of the total *Obesity cohort* served as the standard population, and weights were applied accordingly. The weighted estimates were considered the primary results. Analyses were additionally stratified by the healthcare setting in which the first BMI ≥ 30 kg/m^2^ was recorded (primary care, specialised outpatient care or inpatient care). Healthcare setting was assigned using a hierarchical approach that accounted for uncertainty in BMI recording dates. To identify the healthcare setting of the index BMI, we first searched inpatient episodes, followed by primary care and specialised outpatient care. If no match was identified within the same date, the search was expanded using a ±3‐day window.

We further analysed obesity‐related events, including the first recorded obesity diagnosis (ICD‐10 code E66), OMMs (orlistat [ATC: A08AB01], bupropion/naltrexone [ATC: A08AA62], liraglutide [ATC: A10BJ02; restricted to preparations indicated for obesity treatment]) and bariatric surgery (procedure code JDF). Time from index (first BMI ≥ 30 kg/m^2^) to each event was analysed using the Kaplan–Meier estimator, with time prior to index included for descriptive purposes. Events recorded before index (negative time values) represent individuals who had an obesity‐related event prior to their first recorded BMI ≥ 30 kg/m^2^. All available data in the AROS database were used for event identification, whilst graphical representations were restricted to ±5 years from the index date.

For the clinical outcomes (major adverse cardiovascular events [MACE], cardiovascular death, all‐cause mortality, hospitalisation for heart failure, myocardial infarction and stroke), cumulative incidence was estimated using the Kaplan–Meier estimator, and hazard ratios (HRs) with 95% confidence intervals (CIs) were obtained from Cox proportional hazards models. Hazard ratios were reported for comparisons between the total *Obesity cohort* and the matched *General population cohort*. To account for the clustered data structure arising from matching with replacement, robust standard errors were used when estimating CIs. Results were also presented by obesity class. Similar to the analysis of cardiometabolic comorbidities, age–sex standardisation was applied to cumulative incidence curves because age and sex distributions differed across obesity classes; all classes were weighted to match the age (5‐year strata) and sex distribution of the total *Obesity cohort*. Schoenfeld residuals were used to assess the proportional hazards assumption. Visual inspection suggested minor departures from proportionality during the first year of follow‐up; we determined that the reported hazard ratio is a valid summary of the average effect across the study period.

All statistical analyses were conducted using R version 4.3.3 (R Foundation for Statistical Computing, Vienna, Austria).

## 3 Results

### 3.1 Prevalence of Obesity

**FIGURE 1:**
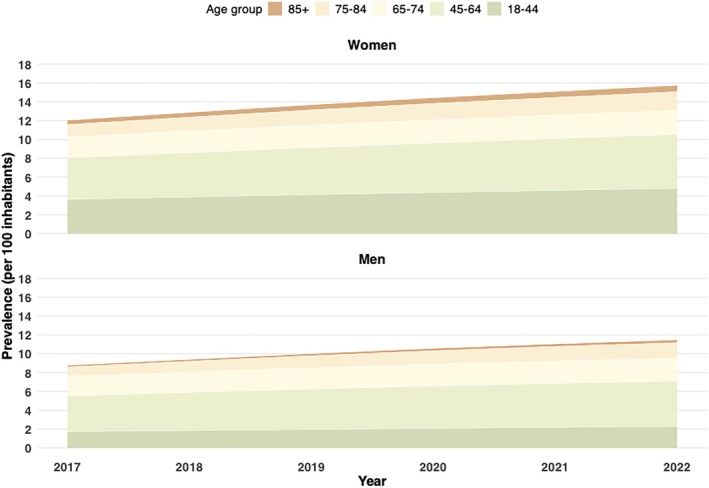
Trends in obesity prevalence by strata of age groups from 2017 to 2022. The selected time period ensures consistent data coverage across regions. Although BMI data in AROS were available from 2013, coverage of BMI recordings varied between regions and over time, likely reflecting differences in the implementation of structured electronic documentation. Data for 2023 were excluded from the prevalence analysis as the calendar year was not complete at the time of data extraction and therefore did not allow for full‐year prevalence estimation.

Between 2017 and 2022, the recorded prevalence of obesity in AROS increased from 10.4% to 13.6% (Figure 1), reaching 15.7% in women and 11.5% in men in 2022.

### 3.2 Baseline Characteristics, Obesity Diagnosis and Comorbidity Patterns

Amongst individuals with available BMI measurements, 328 094 participants had at least one BMI ≥ 30 kg/m^2^ recorded during 2013–2023 (defined as study index) and were included in the *Obesity cohort* for individual‐level analyses (Figure S1). The mean age at index was 53.5 years (SD 17.0), and 55.0% were women. Most participants were classified as obesity class 1 (*n* = 236 601; 72.1%), followed by class 2 (*n* = 63 343; 19.3%) and class 3 (*n* = 28 150; 8.6%). Across obesity classes, 39.7% of first BMI ≥ 30 kg/m^2^ recordings occurred in primary care, whilst 27.7% of participants were first identified in inpatient care (Table 1).

**TABLE 1:** Baseline characteristics of the overall *Obesity cohort* (total and by classes of obesity), including data from all healthcare levels.

	Obesity cohort (all)	Obesity class 1 (BMI 30–34.9)	Obesity class 2 (BMI 35–39.9)	Obesity class 3 (BMI ≥ 40)
Number of patients	328 094	236 601	63 343	28 150
Age at index, mean (SD)	53.5 (17.0)	54.6 (17.0)	51.6 (16.9)	48.4 (16.5)
Age group, *n* (%)				
Age 18–44	101 782 (31.0)	68 011 (28.7)	21 971 (34.7)	11 800 (41.9)
Age 45–64	130 398 (39.7)	94 077 (39.8)	25 399 (40.1)	10 922 (38.8)
Age 65–74	58 949 (18.0)	44 928 (19.0)	10 246 (16.2)	3775 (13.4)
Age 75–84	29 580 (9.0)	23 449 (9.9)	4696 (7.4)	1435 (5.1)
Age 85+	7385 (2.3)	6136 (2.6)	1031 (1.6)	218 (0.8)
Sex, *n* (%)				
Male	147 566 (45.0)	111 469 (47.1)	25 719 (40.6)	10 378 (36.9)
Female	180 528 (55.0)	125 132 (52.9)	37 624 (59.4)	17 772 (63.1)
Region of residence, *n* (%)				
Region of Stockholm	179 312 (54.7)	136 270 (57.6)	30 889 (48.8)	12 153 (43.2)
Region of Skåne	127 535 (38.9)	89 089 (37.7)	26 553 (41.9)	11 893 (42.2)
Region of Dalarna	21 247 (6.5)	11 242 (4.8)	5901 (9.3)	4104 (14.6)
Care level at first observed BMI > = 30 kg/m^2^, *n* (%)				
Primary care	130 399 (39.7)	92 171 (39.0)	26 312 (41.5)	11 916 (42.3)
Specialised outpatient care	77 181 (23.5)	55 965 (23.7)	14 548 (23.0)	6668 (23.7)
Inpatient care	90 916 (27.7)	67 914 (28.7)	16 355 (25.8)	6647 (23.6)
Healthcare level missing	29 598 (9.0)	20 551 (8.7)	6128 (9.7)	2919 (10.4)
BMI at baseline, categorisation of obesity and waist				
BMI at index (kg/m^2^), mean (SD)	33.9 (4.3)	31.8 (1.4)	37.0 (1.4)	44.4 (4.7)
Obesity class 1, *n* (%)	236 601 (72.1)	236 601 (100.0)	0 (0.0)	0 (0.0)
Obesity class 2, *n* (%)	63 343 (19.3)	0 (0.0)	63 343 (100.0)	0 (0.0)
Obesity class 3, *n* (%)	28 150 (8.6)	0 (0.0)	0 (0.0)	28 150 (100.0)
Recorded diagnosis of obesity (E66)	94 522 (28.8)	49 320 (20.8)	27 441 (43.3)	17 761 (63.1)
Waist circumference (cm), median (IQR)	111.0 (104.0–118.5)	108.0 (102.0–114.0)	118.5 (111.0–125.0)	128.0 (119.5–136.5)
Waist circumference missing, *n* (%)	301 835 (92.0)	217 775 (92.0)	58 240 (91.9)	25 820 (91.7)
Cardiometabolic conditions, *n* (%)				
Hypertension (diagnoses and SBP ≥ 140 mmHg)	165 827 (50.5)	120 819 (51.1)	31 478 (49.7)	13 530 (48.1)
Hypertension (diagnoses)	129 554 (39.5)	95 434 (40.3)	24 142 (38.1)	9978 (35.4)
Atrial fibrillation	24 073 (7.3)	17 849 (7.5)	4342 (6.9)	1882 (6.7)
Ischaemic heart disease	32 138 (9.8)	24 875 (10.5)	5465 (8.6)	1798 (6.4)
Heart failure	17 587 (5.4)	12 479 (5.3)	3376 (5.3)	1732 (6.2)
Stroke	12 636 (3.9)	9914 (4.2)	2023 (3.2)	699 (2.5)
PAD	4212 (1.3)	3503 (1.5)	532 (0.8)	177 (0.6)
Diabetes mellitus Type 2	50 101 (15.3)	34 608 (14.6)	10 624 (16.8)	4869 (17.3)
CKD based on diagnoses	13 428 (4.1)	10 162 (4.3)	2255 (3.6)	1011 (3.6)
CKD defined by diagnosed and eGFR < 60 mL/min	43 183 (13.2)	33 039 (14.0)	7326 (11.6)	2818 (10.0)
Dyslipidaemia	133 217 (40.6)	98 473 (41.6)	24 555 (38.8)	10 189 (36.2)
Obstructive sleep apnoea	24 080 (7.3)	15 428 (6.5)	5435 (8.6)	3217 (11.4)
Other comorbidities, *n* (%)				
Cancer	31 192 (9.5)	24 932 (10.5)	4700 (7.4)	1560 (5.5)
MASLD/MASH^a^	2268 (0.7)	1598 (0.7)	477 (0.8)	193 (0.7)
Thromboembolism	19 390 (5.9)	14 100 (6.0)	3564 (5.6)	1726 (6.1)
Arthrosis	66 609 (20.3)	48 994 (20.7)	12 635 (19.9)	4980 (17.7)
Gallbladder surgery	18 616 (5.7)	12 769 (5.4)	4017 (6.3)	1830 (6.5)
Hernia surgery	5869 (1.8)	5066 (2.1)	636 (1.0)	167 (0.6)
Laboratory values^b^				
Total cholesterol (mmol/L), median (IQR)	5.0 (4.2–5.8)	5.0 (4.2–5.8)	4.9 (4.2–5.7)	4.8 (4.2–5.6)
Total cholesterol missing, *n* (%)	202 850 (61.8)	144 173 (60.9)	40 623 (64.1)	18 054 (64.1)
LDL‐C (mmol/L), median (IQR)	3.1 (2.4–3.9)	3.1 (2.4–3.9)	3.1 (2.5–3.9)	3.1 (2.5–3.8)
LDL‐C missing, *n* (%)	209 978 (64.0)	149 841 (63.3)	41 808 (66.0)	18 329 (65.1)
HDL‐C (mmol/L), median (IQR)	1.2 (1.0–1.5)	1.2 (1.0–1.5)	1.2 (1.0–1.4)	1.1 (1.0–1.4)
HDL‐C missing, *n* (%)	210 043 (64.0)	149 972 (63.4)	41 778 (66.0)	18 293 (65.0)
Triglycerides (mmol/L), median (IQR)	1.5 (1.1–2.2)	1.5 (1.1–2.1)	1.6 (1.2–2.2)	1.6 (1.2–2.2)
Triglycerides missing, *n* (%)	247 834 (75.5)	179 112 (75.7)	47 968 (75.7)	20 754 (73.7)
NT‐proBNP (ng/L), median (IQR)	175.0 (62.0–759.0)	180.0 (63.0–769.0)	159.0 (57.0–725.0)	164.0 (57.0–766.2)
NT‐proBNP missing, *n* (%)	287 472 (87.6)	207 067 (87.5)	55 887 (88.2)	24 518 (87.1)
HbA_1c_ (mmol/mol), median (IQR)	40.0 (36.0–50.0)	40.0 (36.0–49.0)	41.0 (36.0–51.0)	41.0 (36.0–50.0)
HbA_1c_ missing, *n* (%)	216 651 (66.0)	156 704 (66.2)	42 122 (66.5)	17 825 (63.3)
Prediabetes (39–47 mmol/mol)^c^, ^d^	24 708 (34.9)	17 572 (34.7)	4676 (35.0)	2460 (35.8)
Prediabetes (42–47 mmol/mol)^d^, ^e^	10 724 (15.1)	7446 (14.7)	2133 (16.0)	1145 (16.7)
eGFR (mL/min), mean (SD)	79.5 (20.0)	78.6 (19.9)	81.1 (20.0)	83.9 (20.4)
eGFR < 60 mL/min, *n* (%)	39 273 (14.9)	30 150 (15.6)	6634 (13.6)	2489 (11.7)
eGFR < 30 mL/min, *n* (%)	5658 (2.2)	4230 (2.2)	995 (2.0)	433 (2.0)
eGFR missing, *n* (%)	65 274 (19.9)	43 937 (18.6)	14 429 (22.8)	6908 (24.5)
Drug utilisation^f^, *n* (%)				
RAASi	106 295 (32.4)	77 211 (32.6)	20 422 (32.2)	8662 (30.8)
RASi	103 719 (31.6)	75 504 (31.9)	19 857 (31.3)	8358 (29.7)
ACE inhibitor	52 215 (15.9)	37 933 (16.0)	9983 (15.8)	4299 (15.3)
Angiotensin‐receptor blocker	55 972 (17.1)	40 822 (17.3)	10 727 (16.9)	4423 (15.7)
Calcium channel blocker	55 143 (16.8)	40 679 (17.2)	10 379 (16.4)	4085 (14.5)
Beta blocker	70 264 (21.4)	51 647 (21.8)	13 154 (20.8)	5463 (19.4)
Lipid lowering treatment (any)	66 937 (20.4)	50 439 (21.3)	12 058 (19.0)	4440 (15.8)
Statins	65 201 (19.9)	49 123 (20.8)	11 738 (18.5)	4340 (15.4)
Ezetimibe	3205 (1.0)	2559 (1.1)	502 (0.8)	144 (0.5)
Platelet inhibitors (any)	41 478 (12.6)	31 580 (13.3)	7262 (11.5)	2636 (9.4)
Acetylsalicylic acid	38 803 (11.8)	29 461 (12.5)	6856 (10.8)	2486 (8.8)
P2Y12 inhibitors	5571 (1.7)	4586 (1.9)	727 (1.1)	258 (0.9)
Anticoagulants (any)	19 880 (6.1)	14 881 (6.3)	3505 (5.5)	1494 (5.3)
Vitamin K inhibitors	10 472 (3.2)	7423 (3.1)	2088 (3.3)	961 (3.4)
Direct oral anticoagulant	9909 (3.0)	7833 (3.3)	1498 (2.4)	578 (2.1)

*Note:* Baseline characteristics for the matched General population cohort are presented in Table S2. Table S2 uses specialist care diagnoses only for comparability across cohorts, because primary care diagnostic data are not available for the General population cohort. See Supporting Informations for detailed definitions.

Abbreviations: ACE, angiotensin converting enzyme; BMI, body mass index; eGFR, estimated glomerular filtration rate; HbA1c, glycated haemoglobin; HDL‐C, high‐density lipoprotein cholesterol; IQR, interquartile range; LDL‐C, low‐density lipoprotein cholesterol; MASLD, Metabolic Dysfunction‐Associated Steatotic Liver Disease; NT‐proBNP, N‐terminal prohormone of brain natriuretic peptide; RAASi, Renin‐angiotensin‐aldosterone system inhibitors; RASi, renin‐angiotensin system inhibitors; SD, standard deviation; SBP, systolic blood pressure.

^a^ Possibly low coverage of this diagnosis in the Swedish clinical practice.

^b^ Most recent value recorded within 3 years prior to the index date.

^c^ HbA_1c_ range classified as pre‐diabetes according to American Diabetes Association (ADA).

^d^ Proportion with prediabetes amongst those without diabetes and with available HbA_1c_ measurements.

^e^ HbA_1c_ range for identification of individuals at high risk for diabetes according to International Expert Committee (IEC).

^f^ Using data recorded within 1 year prior to and including the index date.

**FIGURE 2:**
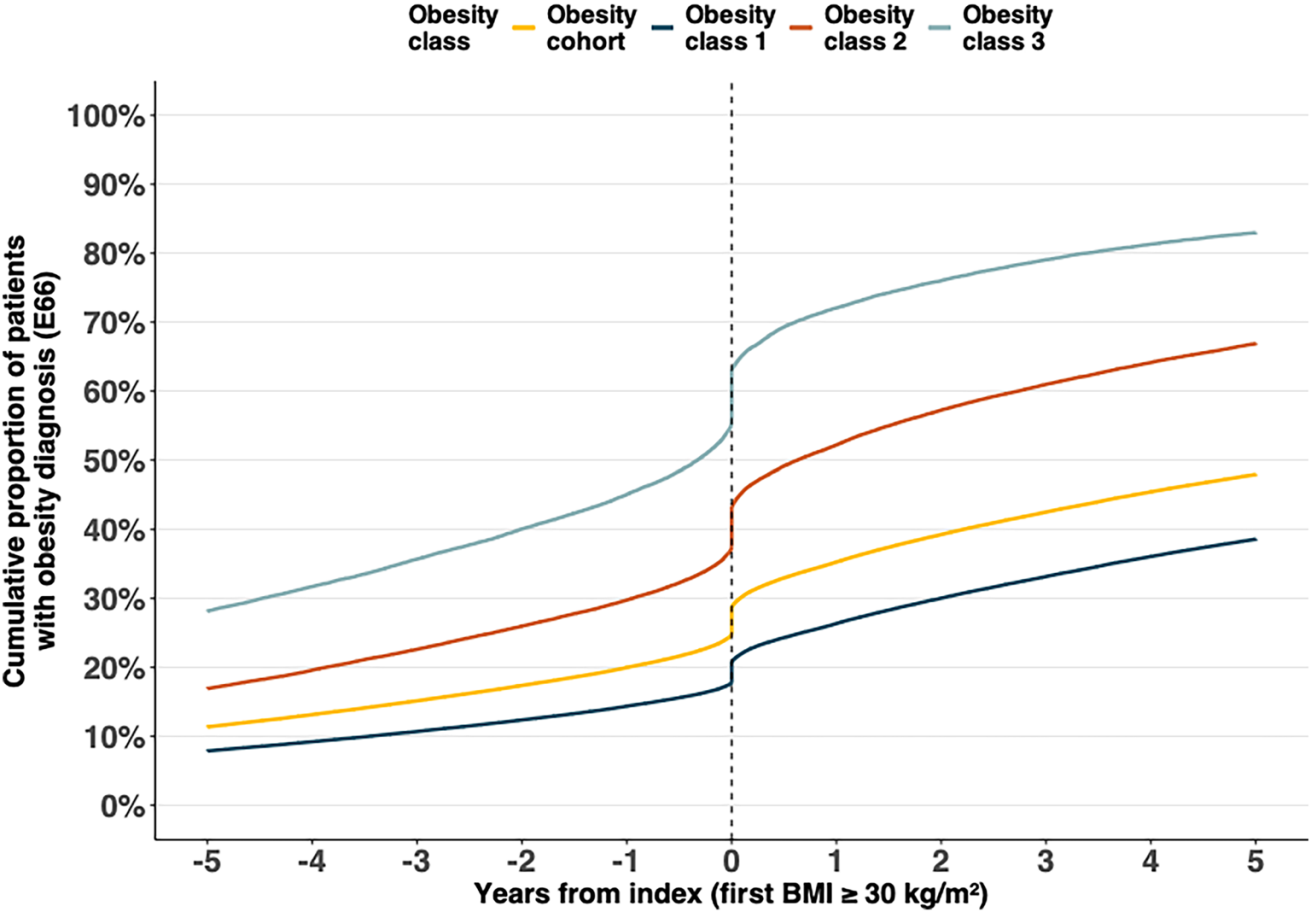
Time to first obesity diagnosis amongst the total *Obesity cohort* and across obesity classes. Cumulative proportions of patients with an obesity diagnosis (ICD‐10 code E66) are shown from 5 years before to 5 years after index (defined as the date of first recorded BMI ≥ 30 kg/m^2^). The sharp increase at study index is expected due to setting of obesity diagnosis at the encounter when BMI ≥ 30 kg/m^2^ is first documented.

At index, 28.8% of all patients had a recorded obesity diagnosis (ICD‐10: E66). For a substantial proportion, the first BMI ≥ 30 kg/m^2^ measurement coincided with the first recorded E66 diagnosis (Figure 2). Five years after index, the cumulative proportion of patients with an obesity diagnosis was 48.0% (95% CI 47.8–48.2), ranging from 38.6% (95% CI 38.4–38.8) in obesity class 1 to 83.0% (95% CI 82.5–83.5) in class 3 (Figure 2).

Comorbidity patterns at index are presented in Table 1. The prevalence of both Type 2 diabetes and prediabetes increased progressively with higher obesity class. Overall, 67.6% had one or more, and 43.5% of patients had two or more cardiometabolic comorbidities at index. In sensitivity analysis restricting baseline comorbidity diagnoses to those recorded ≥ 30 and ≥ 90 days before the index date, the proportion of patients with one or more cardiometabolic comorbidity was 65.6% and 65.2%, respectively (Table S1).

**FIGURE 3:**
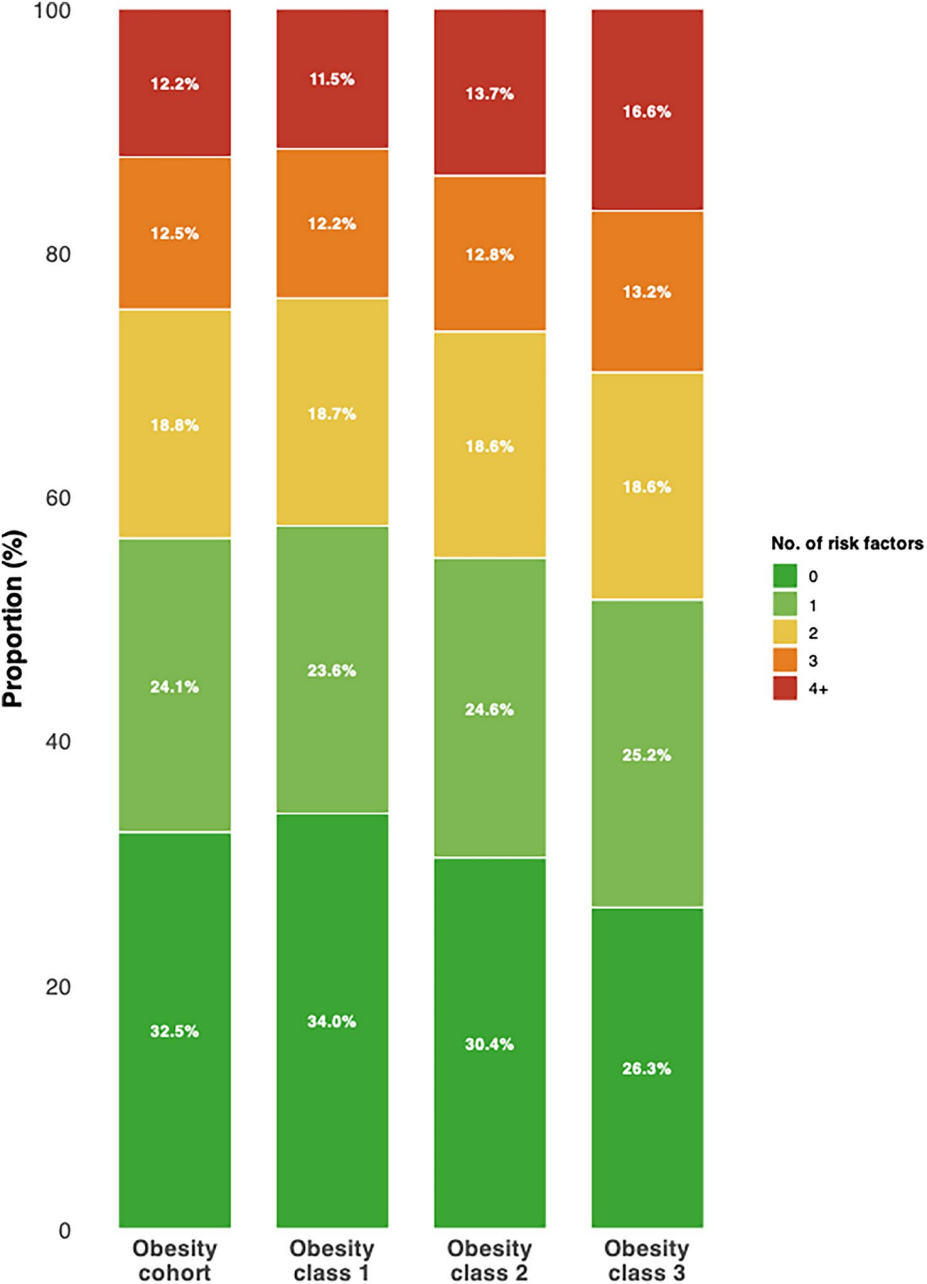
Distribution of the number of cardiometabolic comorbidities (0, 1, 2, 3 or 4+) at the time of first observed BMI ≥ 30 kg/m^2^ across obesity classes. Results are age–sex standardised to the total *Obesity cohort*. Corresponding unstandardised estimates are shown in Figure S2. Comorbidities were defined using ICD‐10 codes, procedure codes, clinical measurements and laboratory data (Supporting Informations).

After age–sex standardisation and stratification by obesity class, the comorbidity burden increased across classes. In obesity class 3, 48.4% of patients had at least two cardiometabolic comorbidities, and 29.8% had three or more (Figure 3). Corresponding unweighted estimates (i.e., without age–sex adjustment) are provided in Figure S2. Comorbidity burden also varied by care setting: amongst patients whose first BMI ≥ 30 kg/m^2^ was recorded in inpatient care, 28.1% had three or more cardiometabolic conditions, compared with 22.8% amongst those identified in primary care (Figure S1).

Across the *Obesity cohort*, the most common cardiometabolic comorbidities were hypertension (50.5%), dyslipidaemia (40.6%) and type 2 diabetes (15.3%; Table 1). When comparing the *Obesity cohort* with the matched *General population cohort* (1 640 464 matched observations corresponding to 887 930 individuals), the burden of cardiometabolic comorbidities was substantially higher across all obesity classes. This comparison was based on specialist‐care diagnoses only, as primary‐care diagnostic data were not available for the *General population cohort* (Table S2).

### 3.3 Obesity Management

**FIGURE 4:**
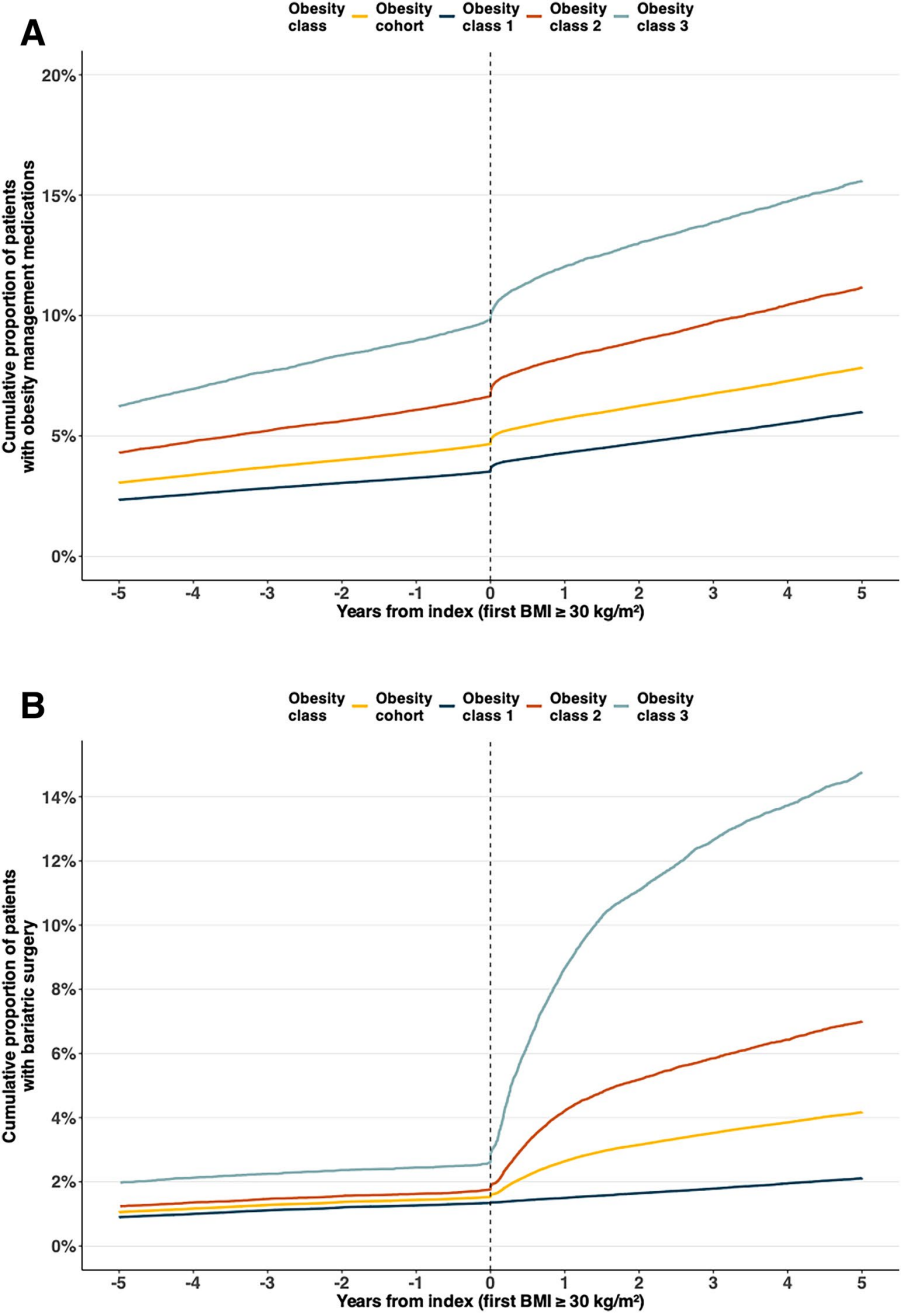
Time to first obesity management treatment amongst the total *Obesity cohort* and across obesity classes. Cumulative proportions of patients are shown 5 years before and after index (first BMI ≥ 30 kg/m^2^ recorded) for pharmacological obesity management medications (A) and bariatric surgery (B).

At index, 4.8% (95% CI 4.7–4.9) of patients across all obesity classes had received an OMM, and 1.6% (95% CI 1.5–1.6) had undergone bariatric surgery. By 5 years after index, 7.8% (95% CI 7.7–7.9) had been treated with an OMM (Figure 4A), and 4.2% (95% CI 4.1–4.2) had received bariatric surgery (Figure 4B). In a sensitivity analysis restricted to patients with index between 2020 and 2023 (*n* = 90 650), the cumulative proportion treated with OMMs was 5.8% at 1 year and 6.4% at 2 years (5.7% and 6.1% in the main analysis, respectively). Treatment uptake was highest in obesity class 3, where 15.6% (95% CI 15.1–16.0) had received an OMM and 14.8% (95% CI 14.3–15.2) had bariatric surgery within 5 years of index.

### 3.4 Clinical Outcomes

**FIGURE 5:**
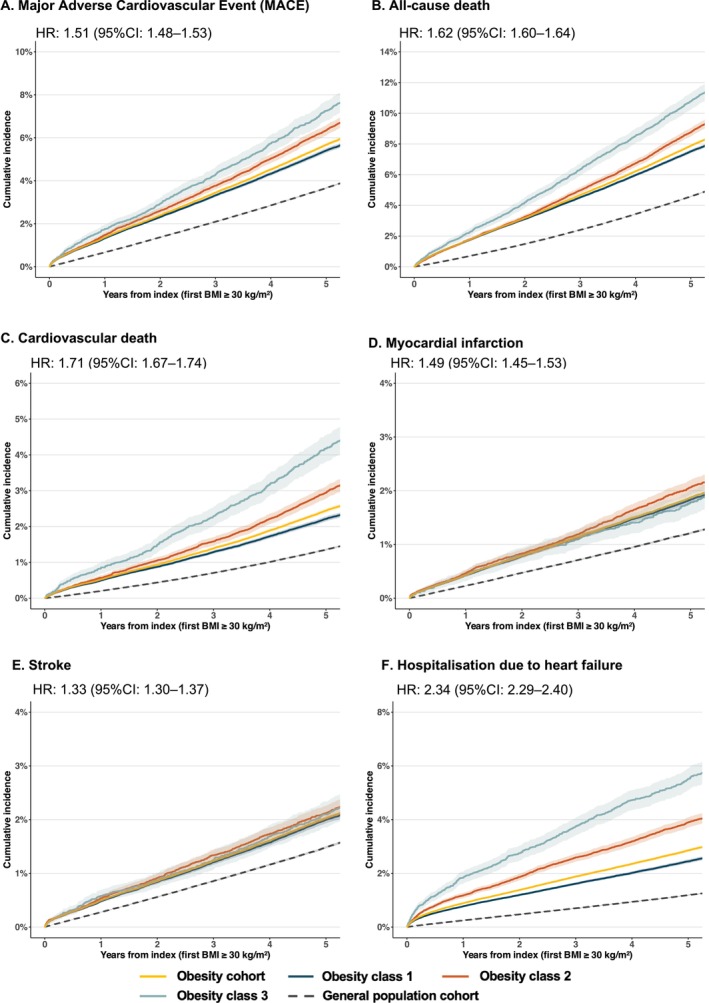
Time‐to–first‐event analyses for adverse cardiovascular outcomes. Cumulative incidence curves, weighted to match the age and sex distribution of the overall Obesity cohort, are presented for: MACE (composite of cardiovascular death, myocardial infarction and stroke) (A); all‐cause mortality (B); cardiovascular death (C); myocardial infarction (D); stroke (E); and hospitalisation for heart failure (F). Hazard ratios represent comparisons between the total Obesity cohort and the General population cohort.

The cumulative incidence of clinical outcomes is shown in Figure 5. Across all outcomes, individuals in the *Obesity cohort* had higher event rates than those in the *General population cohort*. For most outcomes, event rates were progressively higher across obesity classes, although the pattern across obesity classes varied by outcome. The largest relative differences between the *Obesity cohort* and the *General population cohort* were observed for hospitalisation due to heart failure (HR 2.34 [95% CI 2.29–2.40]), cardiovascular death (HR 1.71 [95% CI 1.67–1.74]) and all‐cause death (HR 1.62 [95% CI 1.60–1.64]). No differences were observed across obesity classes for myocardial infarction and stroke.

## 4 Discussion

In this large, retrospective observational study of adults with obesity in Sweden, we found a substantial underutilisation of formal obesity diagnoses in clinical practice and a high burden of cardiometabolic comorbidities at baseline. Only one in four patients had received a documented obesity diagnosis by the time of their first recorded BMI ≥ 30 kg/m^2^, and the proportion remained below 50% after 5 years of follow‐up. Despite the high prevalence of obesity and associated comorbidities, the use of OMMs and surgical obesity treatments was low.

The observed prevalence of obesity increased from 10.4% in 2017 to 13.6% in 2022. This is lower than the estimates reported by the Public Health Agency of Sweden, where survey‐based data indicate a slightly higher prevalence [2]. The age range differs between the Public Health Agency data (16–84 years) and the individuals with obesity in the current study (individuals 18 years or older). However, the discrepancy in prevalence likely reflects differences in population coverage and methodology, as survey‐based estimates include individuals irrespective of healthcare contact, whereas the current analysis is based on recorded BMI values from healthcare visits. Previous research has shown that prevalence estimates derived from diagnostic coding in electronic health records are typically less than half those based on survey data, although the gap narrows with the introduction of automatic BMI codification [16, 17, 18]. In our study, obesity prevalence estimated from recorded BMI values was closer to survey‐based estimates than previous code‐based analyses, suggesting that routine BMI registration in electronic health records may improve the capture of obesity cases in clinical practice. Whilst national surveys only report small sex‐based differences in obesity prevalence [2], women were overrepresented in the obesity cohort in the current study, consistent with earlier findings that women more often seek healthcare [16, 19]. Taken together, the recorded prevalence relative to survey estimates likely reflects a combination of incomplete documentation and the selection of a healthcare‐seeking population.

At the time of the first recorded BMI ≥ 30 kg/m^2^, only one quarter of patients had a documented obesity diagnosis, confirming persistent underdiagnosis in clinical practice [5, 7, 9, 10, 20, 21]. The low level of diagnostic coding helps explain the discrepancy between survey‐ and healthcare data‐based prevalence estimates and emphasises the need for systematic recognition of obesity as a chronic disease within routine care. Experiences from other countries, such as the sharp increase in ICD‐10‐coded obesity after the introduction of automatic coding in Portugal, further illustrate how technical implementation and clinical awareness can improve diagnosis registration [16]. Consistent documentation of BMI and formal obesity diagnoses is essential, as both have been shown to increase the likelihood of receiving obesity‐specific management and support weight‐loss success [5, 7, 8].

Patients with obesity had a high burden of cardiometabolic comorbidities already at the time of their first observed BMI ≥ 30 kg/m^2^, with nearly half presenting with two or more conditions, consistent with previous literature [4, 21, 22]. These findings underscore the close link between obesity and cardiometabolic disease [16, 23, 24]. Despite this, only a small proportion of patients received OMMs or bariatric surgery to lower the risk of complications from excess weight. This limited treatment uptake persisted despite robust evidence supporting the efficacy, safety and cardiometabolic benefits of pharmacological and surgical therapies [25, 26]. However, it is important to note that second‐generation incretin analogues had limited commercial availability and no reimbursement for obesity management in Sweden during the study period, and were not included under OMMs analysed in the current study. The observed OMM uptake therefore reflects only first‐generation agents (orlistat, bupropion/naltrexone and liraglutide), and treatment patterns are likely to change as newer therapies become more widely available. In a sensitivity analysis restricted to patients with a more recent index date between 2020 and 2023, the cumulative proportion treated with an OMM was consistent with the main analysis, confirming that treatment uptake did not materially change during the later study period. Nevertheless, the low uptake of available therapies in the context of a high comorbidity burden highlights an unmet medical need and a treatment gap amongst patients with obesity, pointing to potential for improving patient outcomes through broader adoption of effective obesity management.

Across all obesity classes, most first BMI ≥ 30 kg/m^2^ measurements were observed in primary care, confirming the central role of primary care physicians in early identification of obesity. However, a notable proportion of first BMI recordings occurred in inpatient setting, particularly amongst patients in obesity class 1. This may reflect the older average age and higher comorbidity burden in this group, or that lower BMI levels are more often recorded incidentally during hospitalisations for other acute conditions. These patterns highlight the importance of integrating systematic obesity screening across all healthcare levels.

Our analysis found a substantially higher cardiovascular risk in the obesity population than in the general population, with doubled risk for hospitalisation due to heart failure, and 1.6‐fold higher hazard for all‐cause death. These findings are consistent with broader evidence that obesity confers a higher risk of adverse cardiovascular outcomes. The excess risk generally increased with higher obesity class, with the exception of stroke and myocardial infarction, which did not display differential risks between obesity classes. Several factors may contribute to this observation, including competing risks from conditions with a stronger BMI gradient, such as heart failure and non‐cardiovascular causes of death (cancer, respiratory disease) [27]. In addition, as the analyses were standardised for age and sex only, differences in baseline comorbidity profiles between obesity classes may have influenced the comparisons. The higher incidence of adverse cardiovascular outcomes in patients with obesity emphasises the continued impact of obesity on long‐term health outcomes and reinforces the clinical importance of early diagnosis and management.

Major strengths of this study include the use of comprehensive healthcare data linking national and regional sources, and the inclusion of primary care data capturing routine BMI measurements as well as diagnoses recorded by the primary care physicians. The large sample size and long observation period resulted in a high number of clinical outcomes and treatments available for analysis, allowing detailed characterisation of patients across obesity classes at baseline, and longitudinal assessment of obesity diagnoses, treatment patterns and adverse clinical outcomes In addition, inclusion of age‐ and sex‐matched general population representatives enables further contextualisation of the burden of obesity and its clinical impact on future adverse cardiovascular outcomes and mortality.

However, several limitations should be acknowledged. Although the key patterns of underdiagnosis and undertreatment are consistent with findings from other healthcare settings, the absolute estimates are specific to the Swedish context and may not be directly generalisable to countries with different healthcare systems or coding practices. Lifestyle interventions and referrals to dieticians were not captured. Some comorbidities may have been first recorded around the index date, reflecting the healthcare visit itself, which could overestimate comorbidity burden relative to the general population. To assess whether the reported comorbidity burden was inflated by diagnoses first recorded at the index encounter, we conducted a sensitivity analysis restricting baseline comorbidity diagnoses to those recorded before the index date. The proportion with cardiometabolic comorbidities decreased only modestly, confirming that the majority of comorbidities were established prior to and independently of the index visit. Identification of obesity relied on BMI recordings, which may overestimate obesity prevalence in individuals with high muscle mass. A small proportion of patients had evidence of OMMs or bariatric surgery prior to their first recorded BMI ≥ 30, likely reflecting incomplete historical BMI data due to delayed implementation of electronic health records. In addition, the coverage of bariatric surgery procedures may have decreased in recent years, as an increasing proportion of surgeries are performed within private healthcare settings not fully captured in national registers. This might lead to an underestimation of the number of individuals treated with bariatric surgery (procedure code JDF). The comparison between the *Obesity cohort* and the *General population cohort* was matched on year of birth, sex and region, and did not account for socioeconomic factors or smoking, which were either not available or may act as mediators; the reported hazard ratios should therefore be interpreted as overall differences in outcomes rather than independent effects of obesity. Finally, comorbidities were based on coded diagnoses, which may underestimate disease prevalence.

To conclude, obesity remains substantially underdiagnosed and under‐treated in Swedish clinical practice. Although most cases are first identified in primary care, a significant proportion are first recognised in inpatient care. In addition, those with obesity had a high risk for mortality and of developing adverse cardiovascular outcomes over time, with an almost doubled risk for hospitalisation due to heart failure compared with individuals from the general population. Improved recognition, systematic diagnostic coding and broader use of effective treatments are essential to improve health and reduce the clinical burden of obesity and its associated complications.

## Author Contributions

**V.R.:** conceptualisation (equal), investigation (equal), methodology (supporting), writing – original draught (equal), writing – review and editing (equal). **M.S.:** conceptualisation (equal), investigation (equal), methodology (supporting), writing – original draught (equal), writing – review and editing (equal). **J.D.:** conceptualisation (equal), funding acquisition (equal), writing – original draught (equal), writing – review and editing (equal). **A.E.:** conceptualisation (equal), funding acquisition (equal), writing – original draught (equal), writing – review and editing (equal). **T.C.:** conceptualisation (equal), data curation and formal analysis (lead), investigation (equal), methodology (lead), project administration (supporting), resources (lead), software (lead), validation (lead), visualisation (lead), writing – original draught (equal), writing – review and editing (equal). **M.K.S.:** conceptualisation (equal), investigation (equal), methodology (supporting), resources (lead), writing – original draught (equal), writing – review and editing (equal). **A.N.:** conceptualisation (equal), investigation (equal), methodology (supporting), writing – original draught (equal), writing – review and editing (equal).

## Funding

This work was supported by the Novo Nordisk Scandinavia AB and Uppsala Universitet. The Swedish Heart‐Lung Foundation: 20251279. ALF Medicine, Region Stockholm: FoUI‐1002688. Department of Research and Development, Region Kronoberg.

## Conflicts of Interest

V.R. had research support from Department of Research and Development, Region Kronoberg for her time writing the manuscript. V.R. has received honoraria for expert group participation from Astra Zeneca, Novo Nordisk, and Boehringer Ingelheim. M.S. has received honoraria for lectures from Novo Nordisk. J.D. and A.E. are employees of Novo Nordisk Scandinavia AB. T.C. is an employee and shareholder at Sence Research AB, which is a company in biostatistics and epidemiology that received payment from Novo Nordisk for project management, data management and statistical analysis during the conduct of the study. M.K.S. has received honoraria for lectures from Amgen, AstraZeneca, Boehringer Ingelheim, CSL Vifor and Novo Nordisk, and contributed with expertise to Boehringer Ingelheim, GSK, Novo Nordisk advisory boards. A.N. has received personal honoraria for expert group participation, advisory board meeting, lectures with presentations, during the last 3 years from Astra Zeneca, Novo Nordisk, Bayer and Boehringer Ingelheim. Previously from MSD Sweden and Eli Lilly. A.N. has received foundation for research time from Capio S:t Görans hospital and from the Swedish Heart and Lung foundation.

## Supporting information


**Data S1:** dom70845‐sup‐0001‐supinfo.docx.
**Table S1:** Sensitivity analysis of baseline cardiometabolic comorbidity burden excluding diagnoses recorded within 30 and 90 days of the index date.
**Table S2:** Baseline characteristics of the overall *Obesity cohort* (total and by classes of obesity) and the *General population cohort*, with diagnostic data from primary care excluded.
**Figure S1:** Flowchart of the derivation of the *Obesity cohort and* the matched *General population cohort*.
**Figure S2:** Distribution of the number of cardiometabolic comorbidities at the time of first observed BMI ≥ 30 kg/m^2^ in the *Obesity cohort* (overall and by obesity class), without age–sex standardisation.
**Figure S3:** Distribution of the number of cardiometabolic comorbidities at the time of first BMI ≥ 30 kg/m^2^, by obesity class and healthcare level. Results were age–sex standardised to the age (5‐year bands) and sex distribution of the total *Obesity cohort*, allowing comparison of comorbidity burden across both obesity classes and care settings (independent of differences in age and sex distribution).

## Data Availability

Relevant aggregated data and analytical code for this research project will be made available to Bona Fide Researchers upon reasonable request to MKS, following a legal assessment to ensure data confidentiality. Individual participant‐level data will not be shared. Requests can be made immediately after publication of this Article.
