# Supplementary material for: Underdiagnosed and Undertreated: Obesity and Its Cardiometabolic Burden in Swedish Clinical Practice—Insights From the AROS Database
Source: Diabetes Obes Metab. 2026 May 13;28(8):6787–800. doi: 10.1111/dom.70845 (PMC13341372; doi:10.1111/dom.70845)

Supplementary

**Supplementary Table 1.** Sensitivity analysis of baseline cardiometabolic comorbidity burden excluding diagnoses recorded within 30 and 90 days of the index date.

| Variable | Main analysis (%) | 30 days buffer (%) | 90 days buffer (%) |
| --- | --- | --- | --- |
| >=1 comorbidity | 67.6 | 65.6 | 65.2 |
| >=2 comorbidities | 43.4 | 40.2 | 39.5 |
| 0 comorbidities | 32.5 | 34.4 | 34.8 |
| Hypertension | 50.5 | 48.6 | 48.0 |
| Dyslipidaemia | 40.6 | 40.4 | 40.3 |
| Diabetes mellitus | 17.0 | 14.6 | 14.0 |
| Ischemic Heart Disease | 9.8 | 7.9 | 7.6 |
| Atrial Fibrillation | 7.3 | 5.9 | 5.6 |
| Heart Failure | 5.4 | 4.1 | 3.9 |
| CKD total | 13.2 | 13.0 | 12.9 |
| Stroke | 3.9 | 2.9 | 2.8 |
| Peripheral Artery Disease | 1.3 | 1.1 | 1.0 |
| Sleep apnoea | 7.3 | 6.6 | 6.3 |

Comorbidity definitions were identical to the main analysis. A diagnostic buffer of 30 and 90 days was applied to ICD-10-based comorbidities, restricting diagnoses to those recorded ≥30 or ≥90 days before the index date. Laboratory-based components (SBP ≥140 mmHg, eGFR <60 ml/min, total cholesterol ≥5 mmol/L, LDL ≥3 mmol/L) and medication-based components (lipid-lowering treatment) of composite definitions were unchanged, as these represent objective measurements with independent lookback windows (3 years and 12 months, respectively) and are not susceptible to diagnostic ascertainment bias at the index encounter.

**Supplementary table 2.** Baseline characteristics of the overall *Obesity cohort* (total and by classes of obesity) and the *General population cohort*, with diagnostic data from primary care excluded.

|  | Obesity cohort (all) | Obesity class 1  (BMI 30–34.9) | Obesity class 2  (BMI 35–39.9) | Obesity class 3  (BMI ≥40) | General population  cohort |
| --- | --- | --- | --- | --- | --- |
| Number of patients | 328,094 | 236,601 | 63,343 | 28,150 | 1,640,464 (887,930 individuals) |
| Age at index, mean (SD) | 53.5 (17.0) | 54.6 (17.0) | 51.6 (16.9) | 48.4 (16.5) | 53.5 (17.0) |
| Age group, n (%) |  |  |  |  |  |
| Age 18–44 | 101,782 (31.0) | 68,011 (28.7) | 21,971 (34.7) | 11,800 (41.9) | 508,909 (31.0) |
| Age 45–64 | 130,398 (39.7) | 94,077 (39.8) | 25,399 (40.1) | 10,922 (38.8) | 651,990 (39.7) |
| Age 65–74 | 58,949 (18.0) | 44,928 (19.0) | 10,246 (16.2) | 3,775 (13.4) | 294,745 (18.0) |
| Age 75–84 | 29,580 (9.0) | 23,449 (9.9) | 4,696 (7.4) | 1,435 (5.1) | 147,900 (9.0) |
| Age 85+ | 7,385 (2.3) | 6,136 (2.6) | 1,031 (1.6) | 218 (0.8) | 36,920 (2.3) |
| Sex, n (%) |  |  |  |  |  |
| Male | 147,566 (45.0) | 111,469 (47.1) | 25,719 (40.6) | 10,378 (36.9) | 737,825 (45.0) |
| Female | 180,528 (55.0) | 125,132 (52.9) | 37,624 (59.4) | 17,772 (63.1) | 902,639 (55.0) |
| Region of residence, n (%) |  |  |  |  |  |
| Region of Stockholm | 179,312 (54.7) | 136,270 (57.6) | 30,889 (48.8) | 12,153 (43.2) | 896,559 (54.7) |
| Region of Skåne | 127,535 (38.9) | 89,089 (37.7) | 26,553 (41.9) | 11,893 (42.2) | 637,670 (38.9) |
| Region of Dalarna | 21,247 (6.5) | 11,242 (4.8) | 5,901 (9.3) | 4,104 (14.6) | 106,235 (6.5) |
| BMI at index (kg/m²), mean (SD) | 33.9 (4.3) | 31.8 (1.4) | 37.0 (1.4) | 44.4 (4.7) |  |
| Cardiometabolic conditions, n (%) |  |  |  |  |  |
| Hypertension (diagnoses) | 86,457 (26.4) | 63,436 (26.8) | 16,187 (25.6) | 6,834 (24.3) | 234,708 (14.3) |
| Atrial fibrillation | 22,794 (6.9) | 16,875 (7.1) | 4,115 (6.5) | 1,804 (6.4) | 64,424 (3.9) |
| Ischaemic heart disease | 29,087 (8.9) | 22,518 (9.5) | 4,936 (7.8) | 1,633 (5.8) | 82,116 (5.0) |
| Heart failure | 15,216 (4.6) | 10,779 (4.6) | 2,936 (4.6) | 1,501 (5.3) | 34,640 (2.1) |
| Stroke | 11,696 (3.6) | 9,147 (3.9) | 1,890 (3.0) | 659 (2.3) | 37,770 (2.3) |
| PAD | 3,426 (1.0) | 2,809 (1.2) | 460 (0.7) | 157 (0.6) | 11,855 (0.7) |
| Diabetes Mellitus type 2 | 35,399 (10.8) | 24,366 (10.3) | 7,567 (11.9) | 3,466 (12.3) | 75,355 (4.6) |
| CKD based on diagnoses | 10,707 (3.3) | 8,018 (3.4) | 1,853 (2.9) | 836 (3.0) | 24,799 (1.5) |
| Dyslipidaemia | 72,278 (22.0) | 54,387 (23.0) | 13,064 (20.6) | 4,827 (17.1) | 235,639 (14.4) |
| Obstructive sleep apnoea | 23,074 (7.0) | 14,860 (6.3) | 5,206 (8.2) | 3,008 (10.7) | 48,631 (3.0) |
| Other comorbidities, n (%) |  |  |  |  |  |
| Cancer | 29,749 (9.1) | 23,796 (10.1) | 4,463 (7.0) | 1,490 (5.3) | 113,836 (6.9) |
| MASLD/MASH^a^ | 1,578 (0.5) | 1,083 (0.5) | 342 (0.5) | 153 (0.5) | 2,928 (0.2) |
| Thromboembolism | 15,936 (4.9) | 11,513 (4.9) | 2,961 (4.7) | 1,462 (5.2) | 45,389 (2.8) |
| Arthrosis | 50,933 (15.5) | 37,146 (15.7) | 9,916 (15.7) | 3,871 (13.8) | 168,814 (10.3) |
| Gallbladder surgery | 18,431 (5.6) | 12,639 (5.3) | 3,982 (6.3) | 1,810 (6.4) | 49,452 (3.0) |
| Hernia surgery | 5,842 (1.8) | 5,048 (2.1) | 628 (1.0) | 166 (0.6) | 45,516 (2.8) |
| Drug utilisation^b^, n (%) |  |  |  |  |  |
| RAASi | 106,295 (32.4) | 77,211 (32.6) | 20,422 (32.2) | 8,662 (30.8) | 306,762 (18.7) |
| RASi | 103,719 (31.6) | 75,504 (31.9) | 19,857 (31.3) | 8,358 (29.7) | 299,548 (18.3) |
| ACE inhibitor | 52,215 (15.9) | 37,933 (16.0) | 9,983 (15.8) | 4,299 (15.3) | 151,460 (9.2) |
| Angiotensin-receptor blocker | 55,972 (17.1) | 40,822 (17.3) | 10,727 (16.9) | 4,423 (15.7) | 158,107 (9.6) |
| Calcium channel blocker | 55,143 (16.8) | 40,679 (17.2) | 10,379 (16.4) | 4,085 (14.5) | 157,740 (9.6) |
| Beta blocker | 70,264 (21.4) | 51,647 (21.8) | 13,154 (20.8) | 5,463 (19.4) | 211,745 (12.9) |
| Lipid lowering treatment (any) | 66,937 (20.4) | 50,439 (21.3) | 12,058 (19.0) | 4,440 (15.8) | 220,081 (13.4) |
| Statins | 65,201 (19.9) | 49,123 (20.8) | 11,738 (18.5) | 4,340 (15.4) | 213,922 (13.0) |
| Ezetimibe | 3,205 (1.0) | 2,559 (1.1) | 502 (0.8) | 144 (0.5) | 11,831 (0.7) |
| Platelet inhibitors (any) | 41,478 (12.6) | 31,580 (13.3) | 7,262 (11.5) | 2,636 (9.4) | 141,884 (8.6) |
| Acetylsalicylic acid | 38,803 (11.8) | 29,461 (12.5) | 6,856 (10.8) | 2,486 (8.8) | 130,411 (7.9) |
| P2Y12 inhibitor | 5,571 (1.7) | 4,586 (1.9) | 727 (1.1) | 258 (0.9) | 20,944 (1.3) |
| Anticoagulants (any) | 19,880 (6.1) | 14,881 (6.3) | 3,505 (5.5) | 1,494 (5.3) | 62,106 (3.8) |
| Vitamin K inhibitors | 10,472 (3.2) | 7,423 (3.1) | 2,088 (3.3) | 961 (3.4) | 31,282 (1.9) |
| Direct oral anticoagulant | 9,909 (3.0) | 7,833 (3.3) | 1,498 (2.4) | 578 (2.1) | 32,529 (2.0) |

Abbreviations: ACE, angiotensin converting enzyme; CKD, chronic kidney disease; BMI, body mass index; eGFR, estimated glomerular filtration rate; MASLD, Metabolic Dysfunction-Associated Steatotic Liver Disease; SD, standard deviation.

a Possibly low coverage of this diagnosis in the Swedish clinical practice

b Using data recorded within one year prior to and including the index date

**
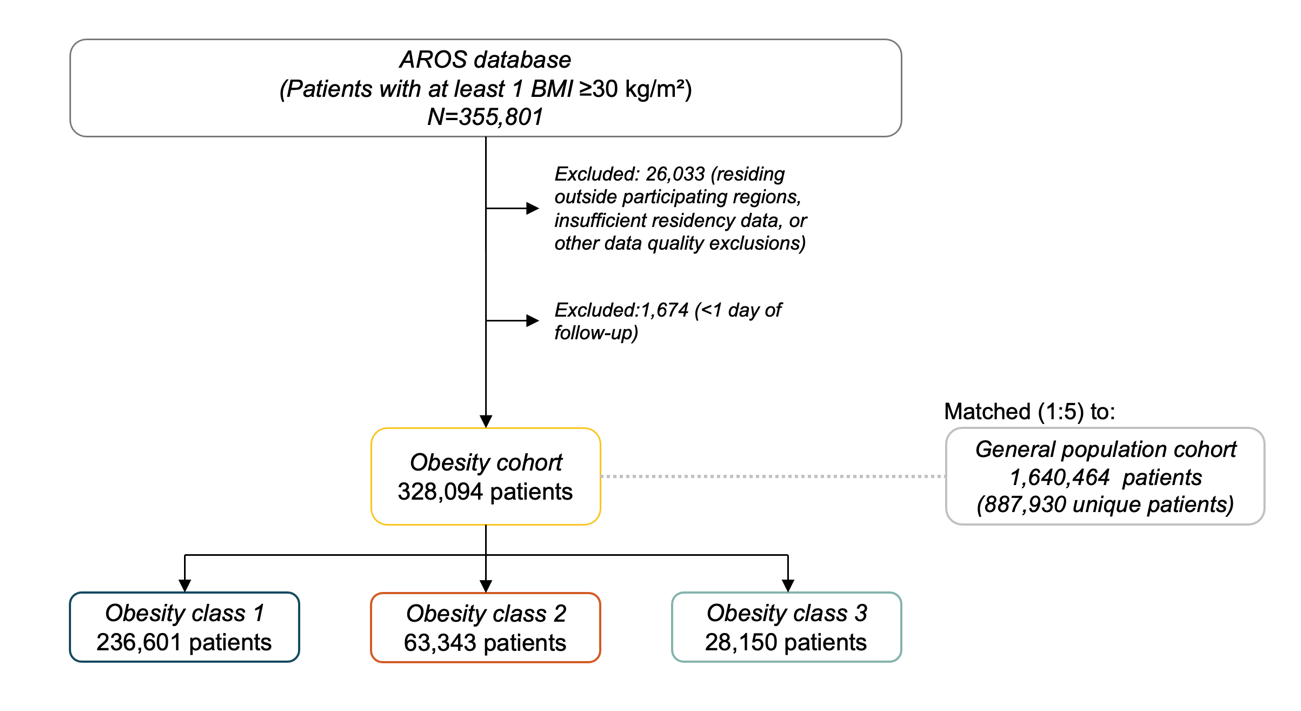
Supplementary figure 1.** Flowchart of the derivation of the *Obesity cohort and* the matched *General population cohort*.

**Supplementary Figure 2.** Distribution of the number of cardiometabolic comorbidities at the time of first observed BMI ≥30 kg/m² in the *Obesity cohort* (overall and by obesity class), without age–sex standardisation.

**Supplementary Figure 3.** Distribution of the number of cardiometabolic comorbidities at the time of first BMI ≥30 kg/m², by obesity class and healthcare level. Results were age–sex standardised to the age (5-year bands) and sex distribution of the total *Obesity cohort,* allowing comparison of comorbidity burden across both obesity classes and care settings (independent of differences in age and sex distribution).

Supplementary methods

**Overview of definitions for cardiometabolic comorbidities and adverse cardiovascular outcomes**

| **Cardiometabolic comorbidity** | **Definition** |
| --- | --- |
| Stroke | **ICD-10:** I60, I61, I63, I64 |
| Peripheral artery disease | **ICD-10:** I70.0, I70.2  **Procedure codes:** PDE, PDF, PDH, PDN, PDQ, PDU, PDW, PEA, PEC, PEE, PEF, PEH, PEN, PEP, PEQ, PER, PET, PEU, PEW, PFA, PFB, PFE, PFH, PFN, PFP, PFQ, PFR, PFT, PFU, PFW, PGH, PGU, PGW, NFQ, NGQ, VH, VJ, VL |
| Chronic kidney disease (CKD) | **ICD-10:** N08.3, N17, N18, N19, E10.2, E11.2, E12.2, E13.2, E14.2, I12.0, I12.9, I13.1, I13.9, Z49.1, Z49.2, Z99.2  *and/or*  an eGFR <60 ml/min based on the most recent measurement recorded within 3 years prior to the index (including the index date). eGFR was calculated from creatinine based on the revised Malmö-Lund formula |
| Diabetes Mellitus | **ICD-10:** E10, E11, E12, E13, E14 |
| Heart failure | **ICD-10:** I50 |
| Ischaemic heart disease | **ICD-10:** I20–I25 |
| Hypertension | **ICD-10:** I10, I11, I12, I13, I15  *and/or*  a systolic blood pressure ≥140 mmHg based on the most recent measurement recorded within 3 years prior to the index (including the index date). |
| Atrial fibrillation | **ICD-10:** I48 |
| Dyslipidaemia | **ICD-10:** E78  *and/or*  a Total cholesterol ≥5.0 mmol/L based on the most recent measurement recorded within 3 years prior to the index (including the index date)  *and/or*  an LDL cholesterol ≥3.0 mmol/L based on the most recent measurement recorded within 3 years prior to the index (including the index date).  *and/or*  a pharmacy dispensation of lipid lowering therapy (ATC: C10) within one year prior to the index (including the index date). |
| Obstructive sleep apnoea | **ICD-10:** G47.3, Z46.4  *and/or*  **Procedure codes:** DG027, DG007 |
| Cancer | **ICD-10:** C (excluding C44) |
| Hernia surgery | **Procedure codes:** JAB |
| Gallbladder surgery | **Procedure codes:** JKA |
| Arthrosis | **ICD-10:** M15–M19 |
| Thromboembolism | **ICD-10:** I26, I80, I82 |
| Metabolic Dysfunction-Associated Steatotic Liver Disease (MASLD) | **ICD-10:** K760 |
| **Adverse Cardiovascular Outcomes** | **Definition** |
| Death due to cardiovascular cause | Death during study period with main cause of death diagnosis code starting with I |
| Hospitalisation due to heart failure | Inpatient hospitalisation with primary diagnosis of I50 |
| MACE | Composite of cardiovascular death (main cause of death diagnosis code starting with I), myocardial infarction (ICD-10: I21), and stroke (ICD-10: I60, I61, I63 and I64) in primary diagnosis position in inpatient care) |
| Myocardial infarction | ICD-10 code of I21 in primary position in inpatient care |
| Stroke | ICD-10 code of I60, I61, I63, I64 in primary position in inpatient care |

**Overview of ATC codes for defining medications**

| **Medication** | **Definition** |
| --- | --- |
| RAASi | ATC: C09, C03DA |
| RASi | ATC: C09 |
| ACE inhibitor | ATC: C09A, C09B |
| Angiotensin-receptor blockers | ATC: C09C, C09D |
| Calcium channel blocker | ATC: C08C |
| Beta blocker | ATC: C07 |
| Lipid-lowering treatment | ATC: C10 |
| Statins | ATC: C10AA |
| Ezetimibe | ATC: C10AX09 |
| Platelet inhibitors | ATC: B01AC |
| Acetylsalicylic acid | ATC: B01AC06 |
| P2Y12 inhibitors | ATC: B01AC04, B01AC22, B01AC24, B01AC25 |
| Anticoagulants | ATC: B01AA, B01AE07, B01AF01, B01AF02, B01AF03 |
| Vitamin K inhibitors | ATC: B01AA |
| Direct oral anticoagulant | ATC: B01AE07, B01AF01, B01AF02, B01AF03 |
| Orlistat | ATC: A08AB01 |
| Bupropion/naltrexone | ATC: A08AA62 |
| Liraglutide | ATC: A10BJ02 (restricted to preparations indicated for obesity treatment) |

*Abbreviations: ACE, angiotensin converting enzyme; ATC, Anatomical Therapeutic Chemical; RAASi, Renin‐angiotensin‐aldosterone system inhibitors; RASi, renin-angiotensin system inhibitors*

**Algorithm for classifying patients as having diabetes mellitus type 1 or type 2**


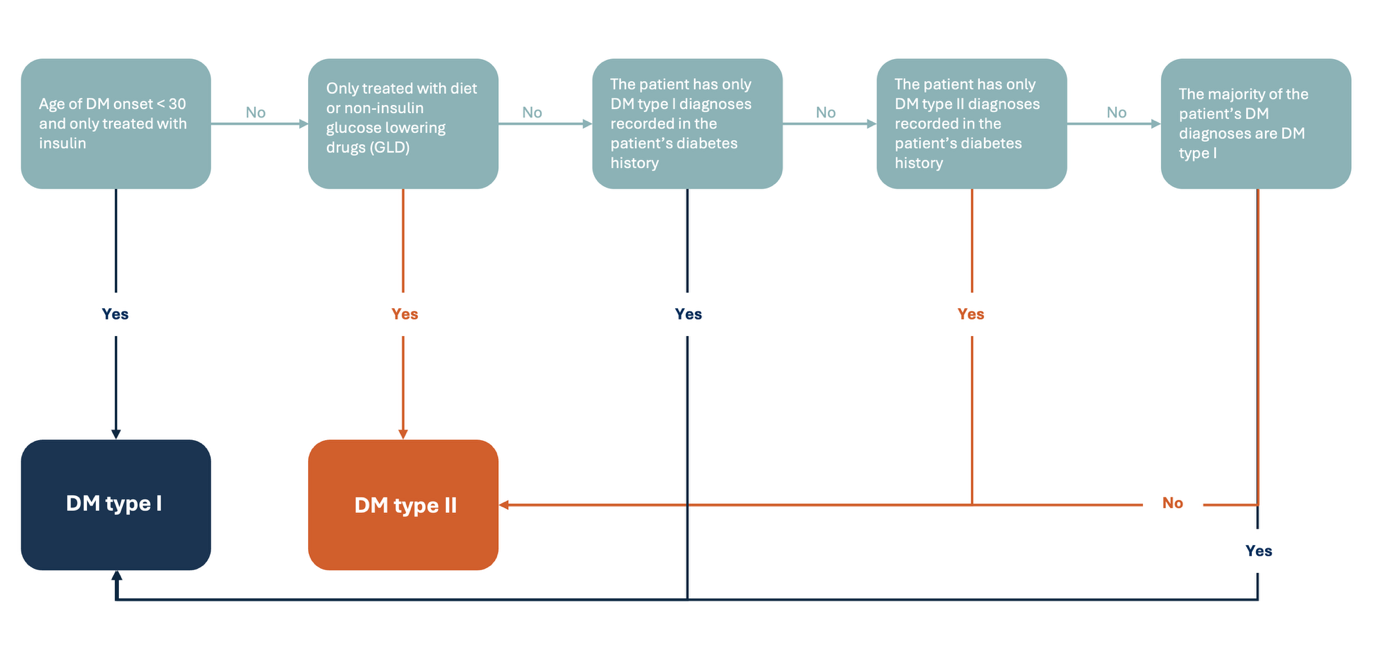

Supplement: Supplementary file 1 — Data S1: dom70845‐sup‐0001‐supinfo.docx. Table S1: Sensitivity analysis of baseline cardiometabolic comorbidity burden excluding diagnoses recorded within 30 and 90 days of the index date. Table S2: Baseline characteristics of the overall Obesity cohort (total and by classes of obesity) and the General population cohort, with diagnostic data from primary care excluded. Figure S1: Flowchart of the derivation of the Obesity cohort and the matched General population cohort. Figure S2: Distribution of the number of cardiometabolic comorbidities at the time of first observed BMI ≥ 30 kg/m2 in the Obesity cohort (overall and by obesity class), without age–sex standardisation. Figure S3: Distribution of the number of cardiometabolic comorbidities at the time of first BMI ≥ 30 kg/m2, by obesity class and healthcare level. Results were age–sex standardised to the age (5‐year bands) and sex distribution of the total Obesity cohort, allowing comparison of comorbidity burden across both obesity classes and care settings (independent of differences in age and sex distribution). [file DOM-28-6787-s001.docx]
